# Supplementary material for: RawHash: enabling fast and accurate real-time analysis of raw nanopore signals for large genomes
Source: Bioinformatics. 2023 Jun 30;39(Suppl 1):i297–307. doi: 10.1093/bioinformatics/btad272 (PMC10311405; doi:10.1093/bioinformatics/btad272)
Supplement: btad272_Supplementary_Data [file btad272_supplementary_data.pdf]

Supplementary Material for RawHash: Enabling Fast and Accurate Real-Time Analysis of Raw Nanopore Signals for Large Genomes

S1. Related Work

To our knowledge, *RawHash* is the first mechanism to efficiently and accurately perform real-time analysis of raw nanopore signals for large genomes. We discuss related work in 1) basecalling, 2) accelerating genome analysis after the basecalling step, and 3) real-time genome analysis with limited computational resources.

Basecalling. Deep learning-based models are utilized by modern basecallers to considerably enhance the precision of identifying a nucleotide base from raw signals compared to traditional non-deep learning-based basecallers [1–6]. Deep learning models can successfully basecall genomes due to the developments and advancements in their architecture, which enables them to model and accurately recognize spatial characteristics in the raw data. Many basecallers have been proposed using modern deep learning-based architectures [7–16] However, the use of complex deep learning models makes basecalling slow and memory-hungry, bottlenecking all genomic analyses that depend on it [6]. Recent works focus on developing methods to speed up the basecalling process. One approach to basecalling acceleration is to use specialized hardware, such as field-programmable gate arrays (FPGAs) [17–21] or processing-in-memory (PIM) [22–24], to perform the basecalling computations. These specialized hardware devices can perform many calculations in parallel, allowing for significant speedups in the basecalling process. Another approach is to use machine learning-based compression techniques to improve the performance of the basecalling process. RUBICON [6] provides a framework to develop hardware-optimized basecallers using neural architecture search [25], knowledge distillation [26], and pruning [27]. Dorado [28], a basecaller by ONT, uses quantization [29] to reduce the bit-width precision at which neural network calculations are performed. All the above works accelerate the basecalling step without eliminating the wasted computation in basecalling. TargetCall [30] proposes a pre-basecalling filter that eliminates the wasted computation in basecalling by leveraging the observation that the majority of reads are discarded after basecalling. However, RawHash is different from these works as its goal is to perform real-time analysis of raw signals without performing the computationally-intensive basecalling step.

Accelerating the genome analysis after basecalling. There are several works that aim to accelerate the entire genome analysis pipeline by accelerating one or multiple steps in the pipeline after basecalling the raw nanopore signals [31, 32]. These works accelerate the pre-alignment filtering and read classification [33–45], chaining [46, 47], read mapping and sequence alignment [48–105] steps. Although these works can significantly improve the performance of the genome analysis pipeline, unlike RawHash, these works cannot perform real-time genome analysis while the raw nanopore signals are generated from nanopore sequencers.

Real-time analysis of raw nanopore signal. Several works perform real-time genome analysis of raw nanopore signals by utilizing adaptive sampling [106–113]. SquiggleFilter [110] uses an ASIC accelerator that quickly filters non-related raw electrical signals before basecalling for viral detection. HARU [112] is an FPGA accelerator that accelerates real-time selective genome sequencing on resource-constrained devices for detecting viral genomes. RawHash differs from these works as it does not require specialized hardware design and can scale to analyze large genomes while matching the throughput of nanopores.

SquiggleNet [111], DeepSelectNet [114], and RawMap [113] require training with machine learning techniques using sequencing reads as training data without using reference genomes. These works train their models to classify raw nanopore signals without mapping them to the reference genome, which is different than RawHash as it maps raw signals to a reference genome. These works often require retraining and reconfiguring the neural network model and architectures. Although such classification approaches can provide high accuracy in labeling reads as target or non-target reads based on a target genome of interest, it can be challenging to easily perform real-time analysis with high accuracy without retraining or reconfiguring these models. RawHash is different than these works as it can map reads to any reference genome using easily configurable parameter settings.

ReadFish [108] and ReadBouncer [115] can scale to mapping reads to large genomes such as a human genome using GPUs or CPUs ((e.g., DeepNano-Blitz [116])) for performing basecalling. Similar to ReadFish and ReadBouncer, RUBRIC [109] use a basecalling approach followed by mapping the basecalled raw signals to analyze raw nanopore signals in real-time. These basecalling approaches are optimized to use the *entire* raw nanopore signal of a read rather than the portions of raw signals produced in real-time, which can be challenging in generating an accurate mapping with a small number of basecalled signals [106, 107]. RawHash differs from ReadFish and ReadBouncer as it does not require powerful computational resources for basecalling, which may not be immediately available for portable sequencers such as ONT MinION. RawHash can directly and accurately map a small number of raw signals (e.g., signals produced in one second) to a reference genome without basecalling them.

We note that ReadFish and ReadBouncer use an interface, *MinKNOW*, required for adaptive sampling in nanopore sequencing. MinKNOW enables tools to analyze the raw nanopore signals and perform adaptive sampling by using functionalities such as Read Until. However, the throughput of these tools using MinKNOW *cannot* exceed the throughput of a nanopore sequencer. Thus, it becomes challenging to fairly compare these tools with the other tools, such as RawHash and Sigmap, for two reasons. First, the throughput of RawHash and Sigmap can be significantly larger than the throughput of a nanopore (Figure 6) due to the lack of support for the MinKNOW interface in these tools. Second, the parameters of RawHash are empirically chosen to provide the best throughput and accuracy without the potential effects of MinKNOW. It is likely that the accuracy of RawHash can improve while providing the same throughput as

a nanopore sequencer. We leave the implementation of MinKNOW for RawHash as future work as well as the comparison of RawHash with ReadFish and ReadBouncer.

UNCALLED [106] and Sigmap [107] are the most relevant works to RawHash. These works map raw nanopore signals to a reference genome without using powerful computational resources (e.g., GPUs), which can be directly used with portable nanopore sequencers. UNCALLED detects events from raw signals, and the probability of k-mers that each event can represent is calculated using k-mer models. UNCALLED identifies the sequence of matching k-mers between the most probable k-mers of events and a reference genome using an FM-index [117]. However, it becomes challenging to accurately identify the matching regions with such a probabilistic model from a large number of matches as the genome size increases [106] (Table 2). Thus, UNCALLED is highly accurate for small genomes (e.g., *E. coli* and *Yeast* genomes) due to the smaller number of probabilistic matches in the reference genome that can be identified accurately.

Sigmap can map raw nanopore signals to genomes larger than the *Yeast* genome (e.g., *Green Algae* with around 100M bases). To achieve this, Sigmap converts the k-mers of the reference genome into events and matches the events between raw nanopore signals with the events of the reference genome. Since events are not necessarily identical when reading the same DNA content, it is challenging to find accurate matches between them due to the signal variations we discuss in Section 2.2. To address this challenge, Sigmap creates a vector from each n consecutive events (i.e., n-dimensional vector space) from the reference genome (i.e., the indexing step) and measures the Euclidean distance between these vectors and the vectors generated from raw nanopore signals (i.e., the mapping step) using a k-d tree structure. Although the distance between vector of events generated from similar regions is close, such a distance calculation is computationally *costly* and suffers from the *curse of dimensionality* that fundamentally prevents accurately and efficiently increasing the number of events within a single vector, which makes it ineffective for larger genomes.

RawHash is different from UNCALLED and Sigmap as it identifies similarities between a reference genome and a raw nanopore signal by efficiently and accurately matching the hash values generated from them without using 1) probabilistic model as proposed in UNCALLED that can be inaccurate for large genomes or costly distance calculations.

S2. Profiling RawHash

S2.1. Profiling the Performance

To analyze the potential bottlenecks and computational overheads in RawHash, we measure the runtime of five steps in RawHash that mainly make up the entire mapping of a read: 1) I/O operations, 2) signal-to-event conversion (Section 2.2), 3) sketching (i.e., quantizing, packing, and hashing events to use them as seeds as described in Sections 2.3 and 2.4), 4) seeding (Section 2.5), and 5) chaining (Section 2.5).

Supplementary Table S1 shows the breakdown analysis of each step in RawHash on various datasets and the overall percentage that both seeding and chaining steps take over the entire runtime of RawHash. We make two key observations. First, we find that the chaining step is the main computational bottleneck in RawHash for all datasets. This is mainly because chaining requires several more computationally costly calculations (e.g., dynamic programming-based computations and sorting) than the other steps. Second, the chaining and seeding steps combined take a larger fraction of the overall runtime as the size of the genome increases (columns in Supplementary Table S1 show increasing genome size from left to right). As the search space increases with larger genomes, the index stores a larger number of seeds for a reference genome. This can increase 1) the time for finding seed matches due to the way hash table structure is implemented in RawHash similar to minimap2 [118] and 2) the number of seed matches per read. The increased number of seed matches can then also increase the time spent in the chaining step as the number of *anchors* (i.e., seed matches in chains) and chains to process increase proportionally.

Table S1: Runtime of the steps in RawHash on various datasets.

	Fraction of entire runtime (%)				
**Tool**	***SARS-CoV-2***	***E. coli***	***Yeast***	***Green Algae***	***Human***
File I/O	0.00	0.00	0.00	0.00	0.00
Signal-to-Event	21.75	1.86	1.01	0.53	0.02
Sketching	0.74	0.06	0.04	0.03	0.00
Seeding	3.86	4.14	3.52	6.70	5.39
Chaining	73.50	93.92	95.42	92.43	94.46
Seeding + Chaining	77.36	98.06	98.94	99.14	99.86

S2.2. Profiling the Chaining Gap Sensitivity

Supplementary Table shows the average length of the gap between a pair of anchors that RawHash finds when mapping raw nanopore signals in various datasets. Read and reference anchors show the average gap length between a pair of anchors found in reads and the reference genome, respectively. We find that the chaining algorithm can tolerate a large number of mismatches and indels especially for larger genomes without significantly sacrificing the mapping accuracy (Table 2).

Table S2: The average gap length between a pair of anchors in reads and a reference genome.

**Tool**	***SARS-CoV-2***	***E. coli***	***Yeast***	***Green Algae***	***Human***
Read Anchors	25.37	42.92	79.31	148.58	200.06
Reference Anchors	20.06	33.39	67.55	127.03	165.44

S3. Runtime and Peak Memory Usage

Supplementary Tables S3 and S4 show the computational resources required by each tool during the indexing and mapping steps, respectively. To measure the required computational resources, we collect CPU time, real time, and peak memory usage of each tool for all the datasets. To collect these results, we use `time -v` command in Linux.

CPU time shows the total user and system time. The real time shows the overall elapsed (i.e., wall clock) time while the application is running. Peak memory usage shows the maximum resident set size in the main memory that the application required to complete its task. We use 32 threads for all applications.

S3.1. Mapping Time per Read

Supplementary Figure S1 shows the average mapping time that each tool spends per read for all the datasets we evaluate.

Table S3: Computational resources required in the indexing step of each tool.

Tool	*Contamination*	*SARS-CoV-2*	*E. coli*	*Yeast*	*Green Algae*	*Human*	*Relative Abundance*
			CPU Time (sec)				
UNCALLED	8.72	9.00	11.08	18.62	285.88	4,148.10	4,382.38
Sigmap	0.02	0.04	8.66	24.57	449.29	36,765.24	40,926.76
RawHash	0.18	0.13	2.62	4.48	34.18	1,184.42	788.88
			Real time (sec)				
UNCALLED	1.01	1.04	2.67	7.79	280.27	4,190.00	4,471.82
Sigmap	0.13	0.25	9.31	25.86	458.46	37,136.61	41,340.16
RawHash	0.14	0.10	1.70	2.06	15.82	278.69	154.68
			Peak memory (GB)				
UNCALLED	0.07	0.07	0.13	0.31	11.96	48.44	47.81
Sigmap	0.01	0.01	0.40	1.04	8.63	227.77	238.32
RawHash	0.01	0.01	0.35	0.76	5.33	83.09	152.80

Table S4: Computational resources required in the mapping step of each tool.

Tool	*Contamination*	*SARS-CoV-2*	*E. coli*	*Yeast*	*Green Algae*	*Human*	*Relative Abundance*
			CPU Time (sec)				
UNCALLED	265,902.26	36,667.26	35,821.14	8,933.52	16,769.09	262,597.83	586,561.54
Sigmap	4,573.18	1,997.84	23,894.70	11,168.96	31,544.55	4,837,058.90	11,027,652.91
RawHash	3,721.62	1,832.56	8,212.17	4,906.70	25,215.23	2,022,521.48	4,738,961.77
			Real time (sec)				
UNCALLED	20,628.57	2,794.76	1,544.68	285.42	2,138.91	8,794.30	19,409.71
Sigmap	6,725.26	3,222.32	2,067.02	1,167.08	2,398.83	158,904.69	361,443.88
RawHash	3,917.49	1,949.53	957.13	215.68	1,804.96	65,411.43	152,280.26
			Peak memory (GB)				
UNCALLED	0.65	0.19	0.52	0.37	0.81	9.46	9.10
Sigmap	111.69	28.26	111.11	14.65	29.18	311.89	489.89
RawHash	4.13	4.20	4.16	4.37	11.75	52.21	55.31

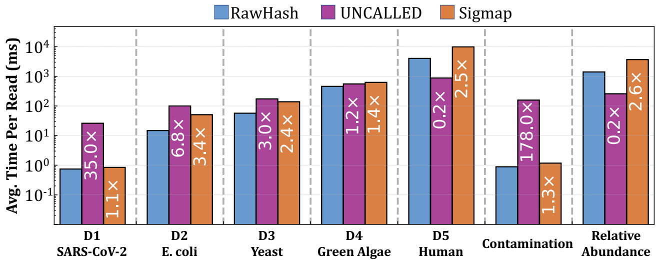

Figure S1: Average time spent per read by each tool in real-time. Values inside the bars show the speedups that RawHash provides over other tools in each dataset.

S4. Limitations of RawHash

We find four limitations of RawHash, which we believe can be improved with further optimizations and better solutions. First, RawHash depends on previously generated k-mer models to generate events from reference genomes. Although these k-mer models can be trained and generated [119, 120], this makes it challenging to adapt the most accurate parameters for each k-mer model based on the nanopore model used for sequencing. A more generic k-mer model that can accurately represent all nanopores is needed to easily adapt RawHash to all possible nanopore models that may be released in the future.

Second, RawHash starts providing lower recall values as the genome size increases, which indicates that a larger portion of correct reads cannot be mapped by RawHash due to the increase in the number of false negatives. Although such an increase in false negatives does not substantially affect some applications, such as contamination analysis, where providing higher precision is more critical to correctly identify the contaminated sample, improving it is useful to provide more accurate genome analysis overall.

Third, we perform our relative abundance estimations based on a priori knowledge of reference genomes. While such an experiment can still be useful in practical scenarios, this is not the common case in metagenomic analysis, where a sample is searched against a significantly larger set of species. We expect that our mechanism can still scale to such metagenomic analyses given that many metagenomic databases are efficiently constructed by including fewer and useful information for each species [121], as opposed to our analysis, where we include whole-genome references.

Fourth, we observe that the throughput of RawHash is expected to reach the throughput of a nanopore when analyzing reference genomes slightly larger than a human genome. Such a limitation can be alleviated by applying 1) seeding techniques that provide faster and more space-efficient searches in large spaces and 2) chaining algorithms that are optimized for hash-based seed matches without the notion of distance between seeds, unlike the chaining algorithm used in Sigmap.

S5. Configuration

S5.1. Parameters

In Supplementary Table S5, we show the parameters of each tool for each dataset. In Supplementary Table S6, we show the details of the preset values that RawHash sets in Supplementary Table S5. For UNCALLED, Sigmap, and minimap2, we use the same parameter setting for all datasets. For the sake of simplicity, we only show the parameters that we explicitly set in each tool. For the descriptions of all the other parameters, we refer to the help message that each tool generates, including RawHash.

We note that the parameter names shown in Supplementary Table S7 are different from the parameters explained in Sections 2.3 and 2.4, although these parameters essentially perform in the same way as explained in these sections, which we describe next. First, the quantization parameter, Q in Section 2.3, is set using the `-q` parameter. Second, the value of p in Section 2.3 (i.e., pruned bits) can be calculated as $p = Q - l - 3$ where l is the least significant l bits of Q. We use l instead of q due to its easier programmability in our tool. This l value is set using the `-l` parameter in RawHash. Third, the number of events packed together, n in Section 2.4, is set using the `-e` parameter.

We set these `-q`, `-l`, and `-e` parameters empirically for three types of datasets: 1) viral genomes, 2) small genomes (i.e., $< 50M$ bases, and 3) large genomes (i.e., $> 50M$ bases) using the preset values `-x viral`, `-x sensitive`, and `-x fast`, respectively. In our empirical analysis, we identify that accuracy and performance are significantly impacted by the values we set for `-e`, `-q` and `-l` for three reasons. First, `e` determines the number of quantized event values packed in a single hash value. Packing a larger number of events improves the sensitivity as it becomes more challenging to find larger consecutive matches of quantized event values than finding a smaller number of consecutive matches. Finding a smaller set of matches can decrease the time spent in seeding and chaining, as we explain in Supplementary Section S2. Second, these values determine the level of quantization of actual event values. Smaller `-q` and `-l` values can lead to loss of information due to storing only fewer bits that cannot be useful for identifying significantly different events. Larger `-q` and `-l` values can generate different quantized values for highly similar event values that may be corresponding to the same DNA content. Third, the number of bits that we store for each event, which are determined by `-q` and `-l`, impacts the number of events that can be packed in a single 32-bit or 64-bit value. Packing a larger number of events in a single hash value directly impacts sensitivity as discussed earlier in this paragraph (first point).

S5.2. Versions

Supplementary Table S7 shows the version and the link to these corresponding versions of each tool that we use in our experiments.

Table S5: Parameters we use in our evaluation for each tool and dataset in mapping.

Tool	*Contamination*	*SARS-CoV-2*	*E. coli*	*Yeast*	*Green Algae*	*Human*	*Relative Abundance*
RawHash	-x viral -t 32	-x viral -t 32	-x sensitive -t 32	-x sensitive -t 32	-x fast -t 32	-x fast -t 32	-x fast -t 32
UNCALLED	map -t 32						
Sigmap	-m -t 32						
Minimap2	-x map-ont -t 32						

Table S6: Corresponding parameters of presets (-x) in RawHash.

Preset (-x)	Corresponding parameters	Usage
viral	-e 5 -q 9 -l 3	Viral genomes
sensitive	-e 6 -q 9 -l 3	Small genomes (i.e., $< 50M$ bases)
fast	-e 7 -q 9 -l 3	Large genomes (i.e., $> 50M$ bases)

Table S7: Versions of each tool.

Tool	Version	Link to the Source Code
RawHash	0.9	`https://github.com/CMU-SAFARI/RawHash/tree/8042b1728e352a28fcc79c2efd80c8b631fe7bac`
UNCALLED	2.2	`https://github.com/skovaka/UNCALLED/tree/74a5d4e5b5d02fb31d6e88926e8a0896dc3475cb`
Sigmap	0.1	`https://github.com/haowenz/sigmap/tree/c9a40483264c9514587a36555b5af48d3f054f6f`
Minimap2	2.24	`https://github.com/lh3/minimap2/releases/tag/v2.24`

Supplementary References

[1] Y.-z. Zhang, A. Akdemir, G. Tremmel, S. Imoto, S. Miyano, T. Shibuya, and R. Yamaguchi, "Nanopore Basecalling from a Perspective of Instance Segmentation," *BMC bioinformatics*, 2020.

[2] R. Dias and A. Torkamani, "Artificial Intelligence in Clinical and Genomic Diagnostics," *Genome medicine*, vol. 11, 2019.

[3] S. L. Amarasinghe, S. Su, X. Dong, L. Zappia, M. E. Ritchie, and Q. Gouil, "Opportunities and Challenges in Long-Read Sequencing Data Analysis," *Genome biology*, vol. 21, 2020.

[4] D. Senol Cali, J. S. Kim, S. Ghose, C. Alkan, and O. Mutlu, "Nanopore Sequencing Technology and Tools for Genome Assembly: Computational Analysis of the Current State, Bottlenecks and Future Directions," *Briefings in Bioinformatics*, vol. 20, Jul. 2019.

[5] F. J. Rang, W. P. Kloosterman, and J. de Ridder, "From Squiggle to Basepair: Computational Approaches for Improving Nanopore Sequencing Read Accuracy," *Genome Biology*, vol. 19, Jul 2018.

[6] G. Singh, M. Alser, A. Khodamoradi, K. Denolf, C. Firtina, M. B. Cavlak, H. Corporaal, and O. Mutlu, "A framework for designing efficient deep learning-based genomic basecallers," *bioRxiv*, 2022.

[7] Oxford Nanopore Technologies, "Bonito, https://github.com/nanoporetech/bonito."

[8] H. Konishi, R. Yamaguchi, K. Yamaguchi, Y. Furukawa, and S. Imoto, "Halcyon: an accurate basecaller exploiting an encoder-decoder model with monotonic attention," *Bioinformatics*, 2021.

[9] N. Huang, F. Nie, P. Ni, F. Luo, and J. Wang, "SACall: A Neural Network Basecaller for Oxford Nanopore Sequencing Data Based on Self-Attention Mechanism," *IEEE/ACM Transactions on Computational Biology and Bioinformatics*, 2020.

[10] Z. Xu, Y. Mai, D. Liu, W. He, X. Lin, C. Xu, L. Zhang, X. Meng, J. Mafofo, W. A. Zaher *et al.*, "Fast-bonito: A Faster Deep Learning Based Basecaller for Nanopore Sequencing," *Artificial Intelligence in the Life Sciences*, vol. 1, 2021.

[11] V. Boža, B. Brejová, and T. Vinař, "DeepNano: Deep recurrent neural networks for base calling in MinION nanopore reads," *PLOS One*, 2017.

[12] Oxford Nanopore Technologies, "Guppy."

[13] P. Perešíni, V. Boža, B. Brejová, and T. Vinař, "Nanopore base calling on the edge," *Bioinformatics*, 2021.

[14] X. Lv, Z. Chen, Y. Lu, and Y. Yang, "An end-to-end Oxford nanopore basecaller using convolution-augmented transformer," in *BIBM*, 2020.

[15] J. Zeng, H. Cai, H. Peng, H. Wang, Y. Zhang, and T. Akutsu, "Causalcall: Nanopore basecalling using a temporal convolutional network," *Frontiers in Genetics*, 2020.

[16] Y.-M. Yeh and Y.-C. Lu, "MSRCall: A multi-scale deep neural network to basecall Oxford nanopore sequences," *Bioinformatics*, 2022.

[17] Z. Wu, K. Hammad, R. Mittmann, S. Magierowski, E. Ghafar-Zadeh, and X. Zhong, "Fpga-based dna basecalling hardware acceleration," in *2018 IEEE 61st International Midwest Symposium on Circuits and Systems (MWSCAS)*. IEEE, 2018.

[18] C. N. Ramachandra, A. Nag, R. Balasubramonion, G. Kalsi, K. Pillai, and S. Subramoney, "ONT-X: An FPGA approach to real-time portable genomic analysis," in *FCCM*, 2021.

[19] K. Hammad, Z. Wu, E. Ghafar-Zadeh, and S. Magierowski, "A scalable hardware accelerator for mobile DNA sequencing," *TVLSI*, 2021.

[20] Z. Wu, K. Hammad, A. Beyene, Y. Dawji, E. Ghafar-Zadeh, and S. Magierowski, "An FPGA implementation of a portable DNA sequencing device based on RISC-V," in *Newcas*, 2022.

[21] Z. Wu, K. Hammad, E. Ghafar-Zadeh, and S. Magierowski, "FPGA-accelerated 3rd generation DNA sequencing," *TBCS*, 2020.

[22] H. Mao, M. Alser, M. Sadrosadati, C. Firtina, A. Baranwal, D. S. Cali, A. Manglik, N. A. Alserr, and O. Mutlu, "Genpip: In-memory acceleration of genome analysis via tight integration of basecalling and read mapping," in *2022 55th IEEE/ACM International Symposium on Microarchitecture (MICRO)*. IEEE, 2022.

[23] Q. Lou, S. C. Janga, and L. Jiang, "Helix: Algorithm/Architecture Co-design for Accelerating Nanopore Genome Base-calling," in *Proceedings of the ACM International Conference on Parallel Architectures and Compilation Techniques*, 2020.

[24] Q. Lou and L. Jiang, "Brawl: A spintronics-based portable basecalling-in-memory architecture for nanopore genome sequencing," *CAL*, 2018.

[25] B. Zoph and Q. V. Le, "Neural Architecture Search with Reinforcement Learning," *arXiv preprint arXiv:1611.01578*, 2016.

[26] C. Buciluǎ, R. Caruana, and A. Niculescu-Mizil, "Model Compression," in *Proceedings of the 12th ACM SIGKDD international conference on Knowledge discovery and data mining*, 2006.

[27] Y. LeCun, J. Denker, and S. Solla, "Optimal Brain Damage," *Advances in neural information processing systems*, vol. 2, 1989.

[28] Oxford Nanopore Technologies, "Dorado, https://github.com/nanoporetech/dorado."

[29] R. M. Gray and D. L. Neuhoff, "Quantization," *IEEE transactions on information theory*, vol. 44, 1998.

[30] M. B. Cavlak, G. Singh, M. Alser, C. Firtina, J. Lindegger, M. Sadrosadati, N. M. Ghiasi, C. Alkan, and O. Mutlu, "Targetcall: Eliminating the wasted computation in basecalling via pre-basecalling filtering," *bioRxiv*, 2022.

[31] M. Alser, J. Lindegger, C. Firtina, N. Almadhoun, H. Mao, G. Singh, J. Gomez-Luna, and O. Mutlu, "From molecules to genomic variations: Accelerating genome analysis via intelligent algorithms and architectures," *Computational and Structural Biotechnology Journal*, vol. 20, Jan. 2022.

[32] M. Alser, J. Rotman, D. Deshpande, K. Taraszka, H. Shi, P. I. Baykal, H. T. Yang, V. Xue, S. Knyazev, B. D. Singer, B. Balliu, D. Koslicki, P. Skums, A. Zelikovsky, C. Alkan, O. Mutlu, and S. Mangul, "Technology dictates algorithms: recent developments in read alignment," *Genome Biology*, vol. 22, Aug. 2021.

[33] H. Xin, D. Lee, F. Hormozdiari, S. Yedkar, O. Mutlu, and C. Alkan, "Accelerating read mapping with fasthash," *BMC Genomics*, 2013.

[34] H. Xin, J. Greth, J. Emmons, G. Pekhimenko, C. Kingsford, C. Alkan, and O. Mutlu, "Shifted Hamming Distance: A fast and accurate simd-friendly filter to accelerate alignment verification in read mapping," *Bioinformatics*, 2015.

[35] M. Alser, H. Hassan, H. Xin, O. Ergin, O. Mutlu, and C. Alkan, "GateKeeper: A new hardware architecture for accelerating pre-alignment in DNA short read mapping," *Bioinformatics*, 2017.

[36] J. S. Kim, D. Senol Cali, H. Xin, D. Lee, S. Ghose, M. Alser, H. Hassan, O. Ergin, C. Alkan, and O. Mutlu, "GRIM-Filter: Fast seed location filtering in DNA read mapping using processing-in-memory technologies," *BMC Genomics*, 2018.

[37] R. Kaplan, L. Yavits, and R. Ginosar, "RASSA: Resistive pre-alignment accelerator for approximate DNA long read mapping," *IEEE Micro*, 2018.

[38] M. Alser, H. Hassan, A. Kumar, O. Mutlu, and C. Alkan, "Shouji: A fast and efficient pre-alignment filter for sequence alignment," *Bioinformatics*, 2019.

[39] M. Alser, T. Shahroodi, J. Gómez-Luna, C. Alkan, and O. Mutlu, "SneakySnake: A fast and accurate universal genome pre-alignment filter for CPUs, GPUs and FPGAs," *Bioinformatics*, 2020.

[40] G. Singh, M. Alser, D. Senol Cali, D. Diamantopoulos, J. Gómez-Luna, H. Corporaal, and O. Mutlu, "FPGA-based near-memory acceleration of modern data-intensive applications," *IEEE Micro*, 2021.

[41] M. Alser, O. Mutlu, and C. Alkan, "MAGNET: Understanding and improving the accuracy of genome pre-Alignment filtering," *arXiv*, 2017.

[42] Z. Bingöl, M. Alser, O. Mutlu, O. Ozturk, and C. Alkan, "GateKeeper-GPU: Fast and accurate pre-alignment filtering in short read mapping," in *IPDPSW*. IEEE, 2021.

[43] M. Khalifa, R. Ben-Hur, R. Ronen, O. Leitersdorf, L. Yavits, and S. Kvatinsky, "FiltPIM: In-memory filter for DNA sequencing," in *ICECS*, 2021.

[44] N. Mansouri Ghiasi, J. Park, H. Mustafa, J. Kim, A. Olgun, A. Gollwitzer, D. Senol Cali, C. Firtina, H. Mao, N. Almadhoun Alserr, R. Ausavarungnirun, N. Vijaykumar, M. Alser, and O. Mutlu, "GenStore: A high-performance in-storage processing system for genome sequence analysis," in *ASPLOS*, 2022.

[45] T. Shahroodi, M. Zahedi, C. Firtina, M. Alser, S. Wong, O. Mutlu, and S. Hamdioui, "Demeter: A fast and energy-efficient food profiler using hyperdimensional computing in memory," *IEEE Access*, 2022.

[46] L. Guo, J. Lau, Z. Ruan, P. Wei, and J. Cong, "Hardware acceleration of long read pairwise overlapping in genome sequencing: a race between FPGA and GPU," in *FCCM*, 2019.

[47] H. Sadasivan, M. Maric, E. Dawson, V. Iyer, J. Israeli, and S. Narayanasamy, "Accelerating Minimap2 for accurate long read alignment on GPUs," *bioRxiv*, 2022.

[48] Y. Chen, B. Schmidt, and D. L. Maskell, "A hybrid short read mapping accelerator," *BMC Bioinformatics*, 2013.

[49] S. K. Khatamifard, Z. Chowdhury, N. Pande, M. Razaviyayn, C. Kim, and U. R. Karpuzcu, "Read mapping near non-volatile memory," *arXiv*, 2017.

[50] Y. Turakhia, G. Bejerano, and W. J. Dally, "Darwin: A genomics co-processor provides up to 15,000x acceleration on long read assembly," in *ASPLOS*, 2018.

[51] S. D. Goenka, Y. Turakhia, B. Paten, and M. Horowitz, "SegAlign: A scalable GPU-based whole genome aligner," in *SC*, 2020.

[52] A. Nag, C. N. Ramachandra, R. Balasubramonian, R. Stutsman, E. Giacomin, H. Kambalasubramanyam, and P.-E. Gaillardon, "GenCache: Leveraging in-Cache operators for efficient sequence alignment," in *MICRO*, 2019.

[53] Q. Aguado-Puig, S. Marco-Sola, J. C. Moure, D. Castells-Rufas, L. Alvarez, A. Espinosa, and M. Moreto, "Accelerating edit-distance sequence alignment on GPU using the wavefront algorithm," *IEEE Access*, 2022.

[54] Q. Aguado-Puig, S. Marco-Sola, J. C. Moure, C. Matzoros, D. Castells-Rufas, A. Espinosa, and M. Moreto, "WFA-GPU: Gap-affine pairwise alignment using GPUs," *bioRxiv*, 2022.

[55] A. Haghi, S. Marco-Sola, L. Alvarez, D. Diamantopoulos, C. Hagleitner, and M. Moreto, "An FPGA accelerator of the wavefront algorithm for genomics pairwise alignment," in *FPL*, 2021.

[56] D. Senol Cali, G. Kalsi, Z. Bingöl, L. Subramanian, C. Firtina, J. Kim, R. Ausavarungnirun, M. Alser, A. Nori, J. Luna *et al.*, "GenASM: A high-performance, low-power approximate string matching acceleration framework for genome sequence analysis," in *MICRO*, 2020.

[57] J. Lindegger, D. S. Cali, M. Alser, J. Gómez-Luna, and O. Mutlu, "Algorithmic improvement and GPU acceleration of the GenASM algorithm," *arXiv*, 2022.

[58] J. Lindegger, D. S. Cali, M. Alser, J. Gómez-Luna, N. M. Ghiasi, and O. Mutlu, "Scrooge: A fast and memory-frugal genomic sequence aligner for CPUs, GPUs, and ASICs," *arXiv*, 2022.

[59] D. Senol Cali, K. Kanellopoulos, J. Lindegger, Z. Bingöl, G. S. Kalsi, Z. Zuo, C. Firtina, M. B. Cavlak, J. Kim, N. M. Ghiasi, G. Singh, J. Gómez-Luna, N. A. Alserr, M. Alser, S. Subramoney, C. Alkan, S. Ghose, and O. Mutlu, "SeGraM: A universal hardware accelerator for genomic sequence-to-graph and sequence-to-sequence mapping," in *ISCA*, 2022.

[60] D. Fujiki, A. Subramaniyan, T. Zhang, Y. Zeng, R. Das, D. Blaauw, and S. Narayanasamy, "GenAx: A genome sequencing accelerator," in *ISCA*, 2018.

[61] A. Madhavan, T. Sherwood, and D. Strukov, "Race Logic: A hardware acceleration for dynamic programming algorithms," *CAN*, 2014.

[62] H. Cheng, Y. Zhang, and Y. Xu, “Bitmapper2: A GPU-accelerated all-mapper based on the sparse Q-gram index,” *TCBB*, 2018.

[63] E. J. Houtgast, V.-M. Sima, K. Bertels, and Z. Al-Ars, “Hardware acceleration of BWA-MEM genomic short read mapping for longer read lengths,” *Computational Biology and Chemistry*, 2018.

[64] E. J. Houtgast, V. Sima, K. Bertels, and Z. AlArs, “An efficient GPU-accelerated implementation of genomic short read mapping with BWA-MEM,” *CAN*, 2017.

[65] A. Zeni, G. Guidi, M. Ellis, N. Ding, M. D. Santambrogio, S. Hofmeyr, A. Buluç, L. Oliker, and K. Yelick, “Logan: High-performance GPU-based X-drop long-read alignment,” in *IPDPS*, 2020.

[66] N. Ahmed, J. Lévy, S. Ren, H. Mushtaq, K. Bertels, and Z. Al-Ars, “GASAL2: A GPU accelerated sequence alignment library for high-throughput NGS data,” *BMC Bioinformatics*, 2019.

[67] T. Nishimura, J. L. Bordim, Y. Ito, and K. Nakano, “Accelerating the Smith-waterman algorithm using bitwise parallel bulk computation technique on GPU,” in *IPDPSW*, 2017.

[68] E. F. de Oliveira Sandes, G. Miranda, X. Martorell, E. Ayguade, G. Teodoro, and A. C. M. Melo, “CUDAlign 4.0: Incremental speculative traceback for exact chromosome-wide alignment in GPU clusters,” *TPDS*, 2016.

[69] Y. Liu and B. Schmidt, “GSWABE: Faster GPU-accelerated sequence alignment with optimal alignment retrieval for short DNA sequences,” *Concurrency and Computation: Practice and Experience*, 2015.

[70] Y. Liu, A. Wirawan, and B. Schmidt, “CUDASW++ 3.0: Accelerating Smith-Waterman protein database search by coupling CPU and GPU SIMD instructions,” *BMC Bioinformatics*, 2013.

[71] Y. Liu, D. L. Maskell, and B. Schmidt, “CUDASW++: Optimizing Smith-Waterman sequence database searches for CUDA-enabled graphics processing units,” *BMC Research Notes*, 2009.

[72] Y. Liu, B. Schmidt, and D. L. Maskell, “CUDASW++ 2.0: Enhanced Smith-Waterman protein database search on CUDA-enabled GPUs based on SIMT and virtualized SIMD abstractions,” *BMC Research Notes*, 2010.

[73] R. Wilton, T. Budavari, B. Langmead, S. J. Wheelan, S. L. Salzberg, and A. S. Szalay, “Arioc: High-throughput read alignment with GPU-accelerated exploration of the seed-and-extend search space,” *PeerJ*, 2015.

[74] A. Goyal, H. J. Kwon, K. Lee, R. Garg, S. Y. Yun, Y. H. Kim, S. Lee, and M. S. Lee, “Ultra-fast next generation human genome sequencing data processing using DRAGEN Bio-IT processor for precision medicine,” *OJGen*, 2017.

[75] Y.-T. Chen, J. Cong, Z. Fang, J. Lei, and P. Wei, “When Spark Meets FPGAs: A case study for next-generation DNA sequencing acceleration,” in *HotCloud*, 2016.

[76] A. F. Laguna, H. Gamaarachchi, X. Yin, M. Niemier, S. Parameswaran, and X. S. Hu, “Seed-and-Vote based in-memory accelerator for DNA read mapping,” in *ICCAD*, 2020.

[77] P. Chen, C. Wang, X. Li, and X. Zhou, “Accelerating the next generation long read mapping with the FPGA-based system,” *TCBB*, 2014.

[78] Y.-L. Chen, B.-Y. Chang, C.-H. Yang, and T.-D. Chiueh, “A high-throughput FPGA accelerator for short-read mapping of the whole human genome,” *TPDS*, 2021.

[79] D. Fujiki, S. Wu, N. Ozog, K. Goliya, D. Blaauw, S. Narayanasamy, and R. Das, “SeedEx: A genome sequencing accelerator for optimal alignments in subminimal space,” in *MICRO*, 2020.

[80] S. S. Banerjee, M. El-Hadedy, J. B. Lim, Z. T. Kalbarczyk, D. Chen, S. S. Lumetta, and R. K. Iyer, “ASAP: Accelerated short-read alignment on programmable hardware,” *TC*, 2019.

[81] X. Fei, Z. Dan, L. Lina, M. Xin, and Z. Chunlei, “FPGASW: Accelerating large-scale Smith–Waterman sequence alignment application with backtracking on FPGA linear systolic array,” *Interdisciplinary Sciences: Computational Life Sciences*, 2018.

[82] H. M. Waidyasooriya and M. Hariyama, “Hardware-acceleration of short-read alignment based on the Burrows-wheeler transform,” *TPDS*, 2015.

[83] X.-Q. Li, G.-M. Tan, and N.-H. Sun, “PIM-Align: A processing-in-memory architecture for FM-Index search algorithm,” *JCST*, 2021.

[84] Y.-T. Chen, J. Cong, J. Lei, and P. Wei, “A novel high-throughput acceleration engine for read alignment,” in *FCCM*, 2015.

[85] E. Rucci, C. Garcia, G. Botella, A. De Giusti, M. Naiouf, and M. Prieto-Matias, “SWIFOLD: Smith-Waterman implementation on FPGA with OpenCL for long DNA sequences,” *BMC Systems Biology*, 2018.

[86] S. Diab, A. Nassereldine, M. Alser, J. Gómez-Luna, O. Mutlu, and I. E. Hajj, “A framework for high-throughput sequence alignment using real processing-in-memory systems,” *arXiv*, 2022.

[87] F. Zokaee, M. Zhang, and L. Jiang, “FindeR: Accelerating FM-index-based exact pattern matching in genomic sequences through ReRAM technology,” in *PACT*, 2019.

[88] S. Angizi, W. Zhang, and D. Fan, “Exploring DNA alignment-in-memory leveraging emerging SOT-MRAM,” in *GLSVLSI*, 2020.

[89] S. Diab, A. Nassereldine, M. Alser, J. G. Luna, O. Mutlu, and I. E. Hajj, “High-throughput pairwise alignment with the wavefront algorithm using processing-in-memory,” *arXiv*, 2022.

[90] W. Huangfu, S. Li, X. Hu, and Y. Xie, “RADAR: A 3D-ReRAM based DNA alignment accelerator architecture,” in *DAC*, 2018.

[91] Z. I. Chowdhury, M. Zabihi, S. K. Khatamifard, Z. Zhao, S. Resch, M. Razaviyayn, J.-P. Wang, S. S. Sapatnekar, and U. R. Karpuzcu, “A DNA read alignment accelerator based on computational RAM,” *JXCDC*, 2020.

[92] L. Li, J. Lin, and Z. Wang, “PipeBSW: A two-stage pipeline structure for banded Smith-Waterman algorithm on FPGA,” in *ISVLSI*, 2021.

[93] L. Wu, D. Bruns-Smith, F. A. Nothaft, Q. Huang, S. Karandikar, J. Le, A. Lin, H. Mao, B. Sweeney, K. Asanović *et al.*, "FPGA accelerated indel realignment in the cloud," in *HPCA*, 2019.

[94] Y. Yan, N. Chaturvedi, and R. Appuswamy, "Accel-Align: a fast sequence mapper and aligner based on the seed–embed–extend method," *BMC Bioinformatics*, 2021.

[95] J. Daily, "Parasail: SIMD C library for global, semi-global, and local pairwise sequence alignments," *BMC Bioinformatics*, 2016.

[96] S. Kalikar, C. Jain, M. Vasimuddin, and S. Misra, "Accelerating minimap2 for long-read sequencing applications on modern CPUs," *Nature Computational Science*, 2022.

[97] S. Marco-Sola, J. C. Moure, M. Moreto, and A. Espinosa, "Fast gap-affine pairwise alignment using the wavefront algorithm," *Bioinformatics*, 2021.

[98] R. Kaplan, L. Yavits, R. Ginosar, and U. Weiser, "A resistive CAM processing-in-storage architecture for DNA sequence alignment," *IEEE Micro*, 2017.

[99] S. K. Khatamifard, Z. Chowdhury, N. Pande, M. Razaviyayn, C. H. Kim, and U. R. Karpuzcu, "GeNVoM: Read mapping near non-volatile memory," *TCBB*, 2021.

[100] F. Chen, L. Song, Y. Chen *et al.*, "PARC: A processing-in-CAM architecture for genomic long read pairwise alignment using ReRAM," in *ASP-DAC*, 2020.

[101] S. Gupta, M. Imani, B. Khaleghi, V. Kumar, and T. Rosing, "RAPID: A reRAM processing in-memory architecture for DNA sequence alignment," in *ISLPED*, 2019.

[102] F. Zokaee, H. R. Zarandi, and L. Jiang, "AligneR: A process-in-Memory architecture for short read alignment in ReRAMs," *CAL*, 2018.

[103] J. M. Eizenga and B. Paten, "Improving the time and space complexity of the WFA algorithm and generalizing its scoring," *bioRxiv*, 2022.

[104] C. Firtina, K. Pillai, G. S. Kalsi, B. Suresh, D. S. Cali, J. Kim, T. Shahroodi, M. B. Cavlak, J. Lindegger, M. Alser, J. G. Luna, S. Subramoney, and O. Mutlu, "Aphmm: Accelerating profile hidden markov models for fast and energy-efficient genome analysis," *arXiv*, Jul. 2022.

[105] S. Marco-Sola, J. M. Eizenga, A. Guarracino, B. Paten, E. Garrison, and M. Moreto, "Optimal gap-affine alignment in O(s) space," *bioRxiv*, 2022.

[106] S. Kovaka, Y. Fan, B. Ni, W. Timp, and M. C. Schatz, "Targeted nanopore sequencing by real-time mapping of raw electrical signal with UNCALLED," *Nature Biotechnology*, vol. 39, Apr. 2021.

[107] H. Zhang, H. Li, C. Jain, H. Cheng, K. F. Au, H. Li, and S. Aluru, "Real-time mapping of nanopore raw signals," *Bioinformatics*, vol. 37, Jul. 2021.

[108] A. Payne, N. Holmes, T. Clarke, R. Munro, B. J. Debebe, and M. Loose, "Readfish enables targeted nanopore sequencing of gigabase-sized genomes," *Nature Biotechnology*, vol. 39, Apr. 2021.

[109] H. S. Edwards, R. Krishnakumar, A. Sinha, S. W. Bird, K. D. Patel, and M. S. Bartsch, "Real-Time Selective Sequencing with RUBRIC: Read Until with Basecall and Reference-Informed Criteria," *Scientific Reports*, vol. 9, Aug. 2019.

[110] T. Dunn, H. Sadasivan, J. Wadden, K. Goliya, K.-Y. Chen, D. Blaauw, R. Das, and S. Narayanasamy, "SquiggleFilter: An accelerator for portable virus detection," in *MICRO*, 2021.

[111] Y. Bao, J. Wadden, J. R. Erb-Downward, P. Ranjan, W. Zhou, T. L. McDonald, R. E. Mills, A. P. Boyle, R. P. Dickson, D. Blaauw, and J. D. Welch, "SquiggleNet: real-time, direct classification of nanopore signals," *Genome Biology*, vol. 22, Oct. 2021.

[112] P. J. Shih, H. Saadat, S. Parameswaran, and H. Gamaarachchi, "Efficient real-time selective genome sequencing on resource-constrained devices," *arXiv*, Nov. 2022.

[113] H. Sadasivan, J. Wadden, K. Goliya, P. Ranjan, R. P. Dickson, D. Blaauw, R. Das, and S. Narayanasamy, "Rapid Real-time Squiggle Classification for Read Until Using RawMap," *bioRxiv*, Jan. 2023.

[114] A. Senanayake, H. Gamaarachchi, D. Herath, and R. Ragel, "DeepSelectNet: deep neural network based selective sequencing for oxford nanopore sequencing," *BMC Bioinformatics*, vol. 24, Jan. 2023.

[115] J.-U. Ulrich, A. Lutfi, K. Rutzen, and B. Y. Renard, "ReadBouncer: precise and scalable adaptive sampling for nanopore sequencing," *Bioinformatics*, vol. 38, Jul. 2022.

[116] V. Boža, P. Perešíni, B. Brejová, and T. Vinař, "DeepNano-blitz: a fast base caller for MinION nanopore sequencers," *Bioinformatics*, vol. 36, Jul. 2020.

[117] P. Ferragina and G. Manzini, "Opportunistic data structures with applications," in *Proceedings 41st Annual Symposium on Foundations of Computer Science*, 2000.

[118] H. Li, "Minimap2: pairwise alignment for nucleotide sequences," *Bioinformatics*, vol. 34, Sep. 2018.

[119] J. T. Simpson, R. E. Workman, P. C. Zuzarte, M. David, L. J. Dursi, and W. Timp, "Detecting DNA cytosine methylation using nanopore sequencing," *Nature Methods*, vol. 14, Apr. 2017.

[120] Oxford Nanopore Technologies, "Nanopolish (K-mer models), https://github.com/jts/nanopolish/tree/r10/etc/r10-models."

[121] F. P. Breitwieser, D. N. Baker, and S. L. Salzberg, "KrakenUniq: confident and fast metagenomics classification using unique k-mer counts," *Genome Biology*, vol. 19, Nov. 2018.
